# Identifying what contraceptive side effects women are told about during counseling using nationally representative PMA Ethiopia 2019 cross-sectional data

**DOI:** 10.1371/journal.pgph.0002111

**Published:** 2024-08-15

**Authors:** Linnea A. Zimmerman, Isabella Sarria, Munir Kassa, Celia Karp, Assefa Seme, Solomon Shiferaw

**Affiliations:** 1 Department of Population, Family, and Reproductive Health, Johns Hopkins Bloomberg School of Public Health, Baltimore, Maryland, United States of America; 2 Federal Ministry of Health, Ethiopia, Addis Ababa, Ethiopia; 3 School of Public Health, Addis Ababa University, Addis Ababa, Ethiopia; PLOS: Public Library of Science, UNITED STATES OF AMERICA

## Abstract

Despite widespread concerns about contraceptive side effects among contraceptive users, few studies explore the specific side effects women are told about during contraceptive counseling. It is thus unclear whether women receive appropriate and sufficient information on side effects they may experience. The objective of this study is to describe which specific side effects of hormonal contraception or copper IUD users are counseled on and identify relevant user characteristics associated with receipt of counseling, using nationally representative cross-sectional data from Ethiopia. Data were collected from a nationally representative sample of women between October and December 2019. Analyses were restricted to 2,039 current users of hormonal contraception (implant, injectable, pill, or emergency contraception) or the copper IUD. Descriptive analyses identified the types and number of side effects, across all methods and by the injectable, implant, and pill. Multinomial regression identified factors associated with receipt of counseling on bleeding changes only, non-bleeding changes only, or both, relative to no counseling on side effects, adjusting for method type, source, and socio-economic characteristics. Less than 10% of users were told of at least one bleeding and non-bleeding side effect. Relative to implant users, injectable and other method users were less likely to be told about bleeding changes only (aRRR: 0.65, 95% CI: 0.46–0.93 and aRRR: 0.31, 95% CI: 0.16–0.61, respectively) and users of other methods were less likely to be told about both a bleeding and non-bleeding change (aRRR: 0.43, 95% CI: 0.19–0.93). Users who received their method from a non-public source were less likely to receive counseling on any kind of side effect and nulliparous women were less likely to be told about both kinds of side effects. There is need to improve counseling on the method specific side effects on which women are counseled, particularly in the private sector.

## Background

Contraceptive use has increased substantially in recent years, reaching a global high of 48% of women age 15–49 using a modern method of contraception in 2019 [1]. At the same time, however, over 160 million women and adolescents are estimated to have unmet need for a method of contraception, i.e. they wish to prevent pregnancy but are not using a modern method of contraception, and more than half live in sub-Saharan Africa [1]. One of the most common reasons cited for contraceptive non-use among women who wish to delay pregnancy is concerns about side-effects and other health issues related to contraceptive use [2]. Contraceptive counseling, “the exchange of information on contraceptive methods”, is an important opportunity to address concerns and provide information about potential side effects [3]. The provision of unbiased, scientifically accurate information supports the rights of women to make informed decisions about their reproductive health, including their choice of contraceptive method [4,5]. Counseling that includes information on potential side effects is effective in increasing the likelihood of method adoption and continuation and in decreasing discontinuation rates [6–8]. Conversely, the provision of inaccurate, incomplete, or biased information can increase dissatisfaction and discontinuation [9], and most importantly, is a violation of women’s right to accurate information, limiting their reproductive autonomy [5,10].

Recent measurement efforts have prioritized assessing counseling on side effects. Specifically, items measuring the receipt of this information were included in the Method Information Index (MII), a three-question measure evaluating whether a woman was told about 1) methods other than the method she chose, 2) side effects, and 3) what to do if she experienced side effects [11]. Higher scores on the MII have been shown to be associated with contraceptive behavior; women who reported being informed about all aspects of the MII were 80% less likely to discontinue, while those informed about any one aspect of the MII were 68% less likely to discontinue contraceptive use [12].

Despite the utility of the MII, however, evidence gaps remain related to the content and comprehensiveness of counseling on side effects. Recent evidence from Pakistan and Uganda published suggests that women are frequently counseled on side effects that are not associated with their chosen method [13], but few studies have quantified these specific side effects. Similarly, while a scoping review of multiple studies confirmed that women frequently report contraceptive-induced menstrual bleeding changes as a major concern [14], recent population-based surveys, including from Ethiopia and Uganda, have identified a wide-range of side effects, including some such as headaches and nausea, that are reported as, or more, frequently than bleeding changes among hormonal contraceptive and copper IUD users [15,16]. Despite the frequency and variety of side-effects that women report, few studies have explored whether women receive counseling for these potential outcomes. Understanding the specific side effects on which women are counseled, whether clinically validated or not, is critical to design counseling tools and training programs that align with side effects women are likely to experience and/or that are of particular concern, such as infertility.

Such efforts can be further improved by identifying whether the comprehensiveness of side effect counseling varies across women. Women who use long-acting methods are more likely to receive all components of the MII [17,18], indicating that method choice may influence the comprehensiveness of counseling services (or vice versa). Research across 25 countries documented that the MII was generally positively associated with wealth and education and was higher in urban relative to rural areas and among women who received services from public, relative to private, providers [18]. Evidence thus suggests that women of more privileged backgrounds and those who are older and parous may be more likely to receive comprehensive information on side effects, but this has not been documented.

Ethiopia is one setting where identifying gaps in contraceptive counseling is of particular importance given recent investments in contraceptive programs and increasing contraceptive use. Modern contraceptive use has increased significantly, from approximately 8% of married women in 2000 [19] to 36% in 2019 [20]. Unmet need remains high, however, with 14% of all woman age 15–49 having an unmet need for contraception [20]. The method mix is largely dominated by the injectable (55% of modern contraception users), but use of the contraceptive implant has grown in recent years, from 16% of the method mix in 2014 to 32% in 2019 [20]. Despite increases in use, recent evidence from Ejigu and colleagues found that quality of counseling services, using the MII, has declined recently, from 39% of modern contraceptive users receiving all three components of the MII in 2015 to 12% in 2019 [21]. Concerns regarding side effects are consistently reported as a main contributor to discontinuation [22–24], and discontinuation rates within one year remain high, exceeding 40% [22]. No studies in Ethiopia, including Ejigu and colleagues, have specifically explored what side effects women are told about, however, which will be informative as the country prioritizes efforts to improve counseling practices.

Our objectives are first to describe overall levels of counseling on specific side effects amongst current hormonal contraceptive or Copper-IUD users and secondly, to explore patterns in receipt of counseling by user characteristics; specifically, we examine whether receipt of counseling on bleeding changes, non-bleeding changes, or receipt of both varies by sociodemographic characteristics, accounting for method type and source.

## Methods

### Ethics statement

All consents were provided as oral consent per guidance from the National Research Ethics Review Guidelines that written consent is not required in areas of low literacy or when data collection does not include invasive procedures (e.g. biospecimen collection) [25]. Additionally, women age 15–17 are considered able to consent from themselves for studies that cover sensitive topics, including reproductive health, and parental consent was not required per the National Research Ethics Review Guidelines. PMA Ethiopia received ethical approval from Addis Ababa University, College of Health Sciences (Ref: AAUMF 01–008) and the Johns Hopkins University Bloomberg School of Public Health Institutional Review Board (FWA00000287).

### Data source

Data came from the PMA Ethiopia 2019 nationally-representative, cross-sectional household survey of women aged 15–49 years. PMA Ethiopia is a survey platform implemented in collaboration between Addis Ababa University, the Federal Ministry of Health of Ethiopia, and Johns Hopkins University. Multistage sampling using probability proportional to size within region and urban/rural strata was used to select 265 enumeration areas (Eas). Between October and December 2019, all households within each EA were listed and 35 households were randomly selected. All women aged 15–49 years who were either usual members of the household or who slept in the household the night before were eligible for the cross-sectional survey. After being informed of study procedures, women provided verbal consent and were interviewed by a trained female interviewer. A total of 8,837 women were interviewed.

Eligible women were approached data collectors who explained study procedures and administered oral informed consent. Interviews were conducted on smartphones and uploaded onto an encrypted cloud server, where they were downloaded daily and reviewed for completeness. Patterns in missingness, non-response, and suspicious data patterns were identified and communicated to supervisors to provide clarification and on-the-ground training to enumerators. Additionally, 10% of households within each EA were randomly selected for re-interview and patterns in responses were compared.

Personally identifiable information collected during data collection were available to the data management team and PIs during data collection, however, all identifiable information was deleted from all datasets prior to data being available for analysis. More information about the study design, quality assurance, and ethical considerations are available from the study protocol [26].

### Measures

Our outcome measures explored the specific side effects, number of side effects, and types of side effects on which women were counseled during the visit at which they received their current method. We identified the specific side effects using the question, *“According to the provider*, *what are the possible side effects or problems related to use of [current method]”*, where “current method” indicates the current method used by the woman. Answer options included known side effects, such as changes to menstrual bleeding, in addition to side effects that are reported by women, but which are not clinically validated, such as impacts on fertility [15,16,27]. Answers were coded based on spontaneous response and were not read aloud. After first exploring the list of all side effects, we evaluated the number of side effects on which women reported being counseled. Finally, we constructed a four-category variable exploring whether women were counseled on contraceptive-induced bleeding changes only, non-bleeding changes, both, or neither. Table 1 indicates how side effects were grouped.

**Table 1:** Sample characteristics of users of hormonal contraception and the IUD; PMA Ethiopia Cross-section 2019.

		Total	
		n (unweighted)	% (weighted)
Method			
	Implant	675	33.2
	Injectable	1114	57.4
	Pill	158	6.5
	Other	73	2.9
Source of Method			
	Hospital/health center	1103	53.4
	Health post/HEW	464	27.0
	Private	472	19.6
Age			
	15–19	160	9.0
	20–24	439	21.4
	25–29	578	27.4
	30–34	372	16.8
	35–39	288	14.3
	40–44	160	9.1
	45–49	42	2.2
Marital Status			
	Unmarried	202	8.4
	Married	1837	91.6
Parity			
	0	238	10.8
	1–2	945	44.2
	3+	855	45.0
Education			
	None	648	36.4
	Primary	790	39.6
	Secondary +	601	23.9
Residence			
	Rural	1100	64.1
	Urban	939	35.9
Region			
	Oromiya	449	38.8
	Amhara	451	27.7
	Addis	206	6.4
	SNNP	407	19.4
	Tigray	243	5.2
	Other	283	2.5
Wealth			
	Lowest	241	15.0
	Lower	305	18.3
	Middle	360	21.3
	Higher	421	19.9
	Highest	712	25.5

For regression analyses, described further below, we included socio-demographic characteristics identified from previous literature related to receiving more comprehensive counseling [17,18,21], specifically age (categorical in five year age groups), parity (categorized as 0, 1–2, 3+ births), highest level of schooling attended (none, primary, secondary and above), residence (urban versus rural), and wealth quintile. Recent evidence has also highlighted critical differences in the MII across regions and by method source in Ethiopia [21] and we thus included region (Oromiya, Amhara, Addis, Southern Nations, Nationalities, and Peoples Region (SNNP-R), Tigray, and all other regions) and method source (hospital or health center, health post or HEW, private facilities and pharmacies). Of note, in 2020, a new region, Sidama, was formed from within SNNP-R. Data were collected prior to the creation of this region. Additionally, civil conflict began in Tigray in 2020, leading to a protracted humanitarian emergency. Data were collected prior to the onset of this conflict and are not likely to represent the current situation. Method source was self-reported from users of contraception using the question *“You first started using [current method] in [date]*. *Where did you or your partner get it at that time*?*”*

### Analyses

#### Analytic sample and analysis

Our analytic sample was restricted to women who slept in the household the night before, completed the female questionnaire, and were current users of a modern method of hormonal contraception (implant, injectable, pill, or emergency contraception) or the copper IUD (n = 2,039). Current users were identified using the question *“Are you or your partner currently doing something or using any method to delay or avoid getting pregnant*?*”* and the type of method was assessed using the question *“Which method or methods are you using*?*”*. These are the standard questions used to assess contraceptive use in household-based surveys [28].

To explore patterns of receipt of counseling of specific side effects (Objective 1), descriptive statistics of frequencies and percentages were used to describe the kind and number of side effects on which women were counseled, among all hormonal contraception and copper IUD users and stratified by the three most-commonly used methods (implant, injectable, pill). Sample size limitations prevented stratification by EC or the copper IUD. For Objective 2, we aimed to explore the relationship between receipt of specific side effect counseling and women’s sociodemographic characteristics. Due to sample size limitations, we could not explore each side effect individually, so side effects were grouped as described in the measures section above, generating a categorical variable. As there was no inherent order to the categorical outcome, we used multinomial logistic regression, accounting for clustering within enumeration areas, to estimate the probability of group membership (in this case, receipt of type of side effect counseling) accounting for adjustment variables [29]. We used unadjusted and adjusted multinomial logistic regression models, using women who received no side effect counseling as the reference. Due to high correlation between age and parity (ρ = 0.70), and wealth and residence (ρ = 0.73), we do not include age or wealth in the adjusted regression model. Additionally, as marriage is almost universal in the sample (Table 1), we did not adjust for marital status. Due to sample size limitations, we combine pill, EC, and copper IUD users into one “other” method type. Analyses accounted for complex survey design through application of weights and adjustment for clustering and were conducted using Stata SE 16.1 [30].

## Results

Sociodemographic characteristics of users are shown in Table 1. The majority of current users of hormonal contraception or the IUD used injectables (57.4%), followed by the implant (33.2%). Most received their method from a public hospital or health center (53.4%), though approximately one in five reported receiving their method from a private facility (19.6%). Almost all of the sample (91.6%) was married and had at least one child and approximately two in three women lived in rural areas.

**Fig 1:**
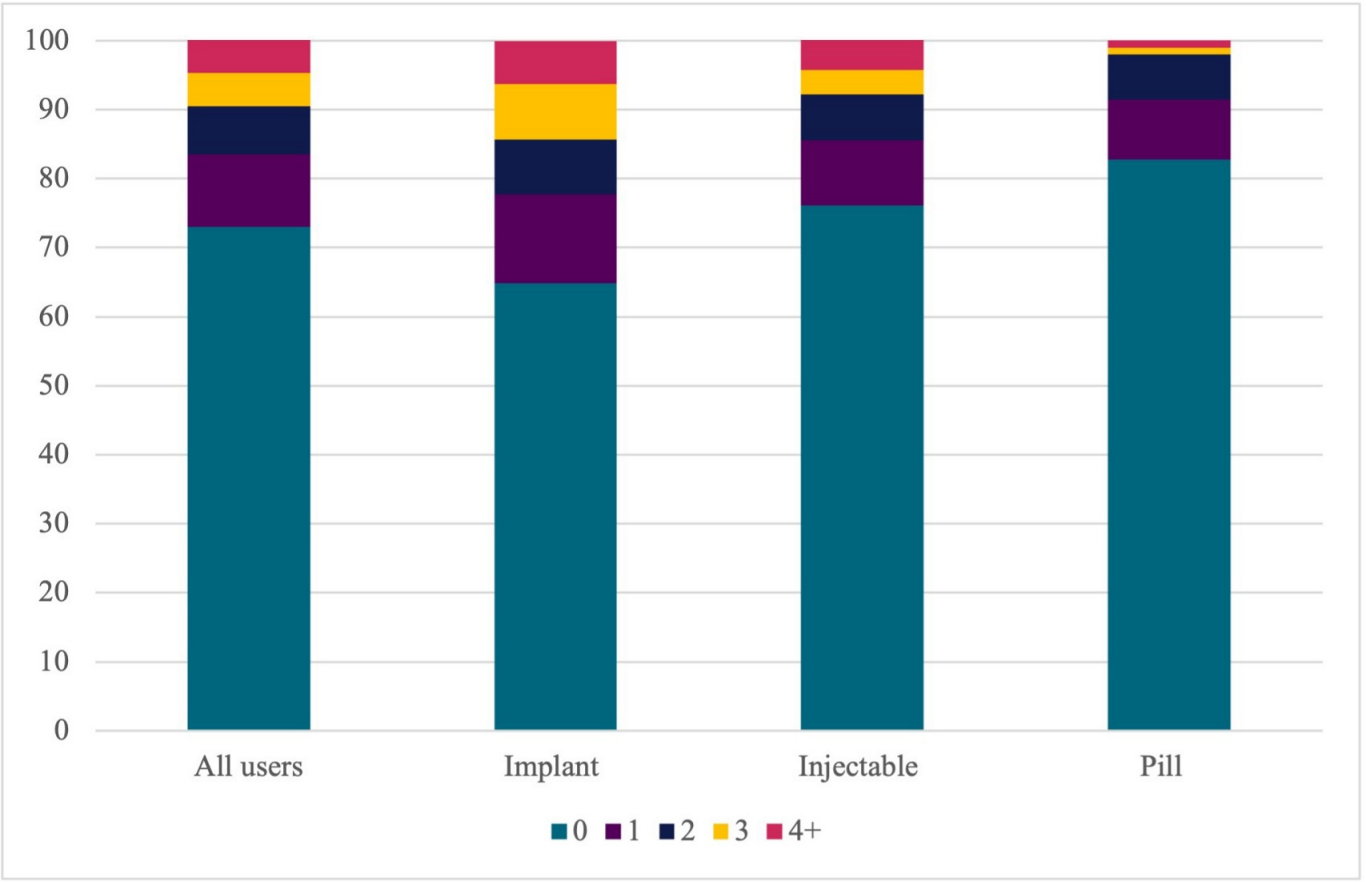
Percentage of women who were told about 0, 1, 2, 3, or 4 plus side effects among current hormonal contraceptive or copper IUD users.

Fig 1 below shows the number of side effects on which women were counseled, across all users and by method. Across all users, almost three in four women reported not receiving counseling on any side effects. Among women who did receive counseling, most received counseling on only one side effect. A higher percentage of implant users reported receiving any counseling on side effects (35.2%) and on more than one side effect (20.2%) than injectable (23.9% and 14.5%, respectively) or pill users (17.2% and 8.5%, respectively). Pill users received the least amount of side effect counseling, relative to implant and injectable users.

Table 2 shows the percentage of users who reported being told about each of the side effects, shown among all current hormonal users and by implant, injectable, and pill users. The most common side effects on which women were counseled were related to menstrual bleeding changes. Fewer than ten percent of women reported receiving counseling about any other potential side effects.

**Table 2:** Percentage of current hormonal contraception or copper IUD users who received counseling on each side effect.

	All current users	Implant users	Injectable users	Pill users
**N**	**2039**	**675**	**1114**	**158**
	**%**	**%**	**%**	**%**
**Bleeding changes**				
Less or No Bleeding	12.7	15.7	12.5	1.7
More Bleeding	10.7	15.3	8.8	4.2
Irregular Bleeding	8.8	12.8	7.2	2.4
Spotting	3.7	5.6	2.7	0.9
General Bleeding (non-specific)	2.6	3.5	2.3	0.9
**Non-bleeding changes**				
Abdominal Pain	0.9	0.8	1.0	0.6
Gain Weight	3.7	4.6	3.5	1.7
Lose Weight	3.7	4.4	3.7	2.3
Acne	1.4	1.0	1.6	1.8
Headache	5.8	9.6	3.8	4.3
Infection	0.4	0.7	0.3	0.6
Nausea	1.0	1.5	0.6	1.6
Menstrual Cramp	1.0	0.1	1.4	2.4
Lower sex drive	0.3	0.3	0.3	0.0
Vaginal dryness	0.2	0.1	0.2	0.0
Infertility/Sterility	1.0	0.1	1.5	0.8
Delayed Fertility	1.1	0.2	1.7	1.3
Method Lost	0.3	0.7	0.1	0.0
Weakness	2.1	3.4	1.4	0.0

**Fig 2:**
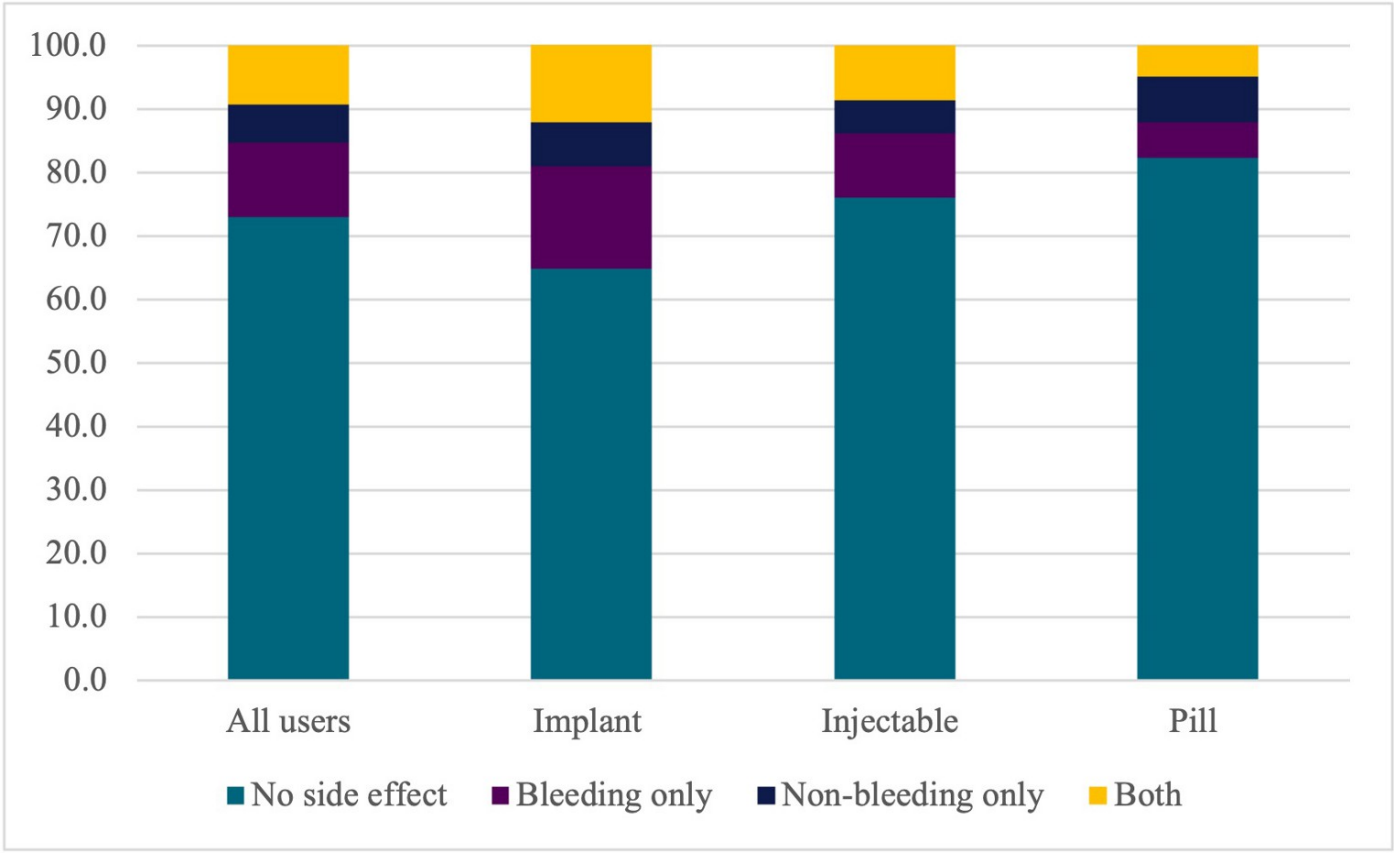
Percentage of women who were told about different kinds of side effects, by method type.

Fig 2 below shows the distribution of types of side effects on which women were counseled. About 20% of all users were counseled on at least one contraceptive-induced menstrual bleeding side effect and about 15% were counseled on a non-bleeding side effect. A higher percentage of implant users (12.9%) reported being told about at least one bleeding and one non-bleeding side effect, but less than five percent of either injectable or pill users reported receiving counseling on both types of side effects.

Unadjusted results assessing the relationship between method choice, selected sociodemographic and environmental factors, and receipt of type of side effect counseling are shown in Table 3 below. Injectable and “other” method users (pill, EC, and copper IUD) were significantly less likely to be told only about a bleeding change (RRR: 0.53, 95% CI: 0.37–0.75 and RRR: 0.27, 95% CI: 0.14–0.52, respectively) and significantly less likely to be told about both a bleeding change and a non-bleeding side effect (RRR: 0.62, 95% CI: 0.40–0.94 and RRR: 0.33, 95% CI: 0.16–0.68, respectively). Parous women were significantly more likely to receive counseling on at least one bleeding and non-bleeding side effect than nulliparous women. Women who received their method from a private source were significantly less likely to receive counseling on bleeding changes only or on both bleeding changes and non-bleeding side effects (RRR: 0.42, 95% CI: 0.26–0.69 and RRR: 0.23, 95% CI: 0.12–0.44, respectively) while women who received services from a health post or HEW were significantly less likely to receive counseling on at least one of each type of side effect than women who received services from a hospital or health center (RRR: 0.59, 95% CI: 0.36–0.98). Region, wealth, and education appeared to be largely unrelated to the type of side effect on which women were counseled, with some exceptions.

**Table 3:** Unadjusted multinomial logistic regression results identifying the relative risk of receiving counseling on Contraceptive Induced Menstrual Bleeding Changes (CIMBC) only, receiving counseling on non-CIMBC only, or on receiving counseling on both types of side effects, relative to receiving no counseling on side effects. PMA Ethiopia 2019 cross-sectional survey.

		CIMBC only			non-CIMBC only			Both		
		RRR	95% CI		RRR	95% CI		RRR	95% CI	
**Method (ref: implant)**										
	Injectable	0.53	0.37	0.75	0.63	0.38	1.06	0.62	0.40	0.94
	Other	0.27	0.14	0.52	0.82	0.45	1.50	0.33	0.16	0.68
**Age (ref: 15–19)**										
	20–24	0.75	0.35	1.64	0.80	0.33	1.93	1.79	0.68	4.72
	25–29	1.12	0.58	2.16	1.35	0.59	3.08	2.86	1.11	7.38
	30–34	1.17	0.57	2.43	0.66	0.26	1.67	2.19	0.85	5.64
	35–39	1.04	0.57	1.90	0.93	0.36	2.39	1.68	0.64	4.41
	40–44	0.78	0.32	1.93	1.02	0.38	2.70	2.51	0.82	7.66
	45–49	0.81	0.27	2.47	0.57	0.08	3.97	2.08	0.48	9.01
**Parity (ref: 0)**										
	1–2	1.84	0.92	3.67	1.10	0.54	2.23	3.05	1.49	6.23
	3+	1.69	0.84	3.42	1.11	0.53	2.33	2.47	1.16	5.24
**Education (ref: None)**										
	Primary	1.13	0.78	1.64	1.19	0.72	1.98	1.05	0.69	1.58
	Secondary	1.38	0.93	2.05	1.63	0.90	2.95	1.73	1.06	2.83
**Residence (ref: rural)**										
	Urban	1.09	0.74	1.61	1.53	0.92	2.56	1.03	0.67	1.59
**Region (ref: Oromiya)**										
	Tigray	0.38	0.23	0.64	0.94	0.47	1.89	0.81	0.47	1.39
	Amhara	0.95	0.56	1.62	1.72	0.83	3.56	1.15	0.65	2.03
	Addis	0.62	0.33	1.16	0.76	0.35	1.68	1.14	0.60	2.16
	SNNP	0.74	0.37	1.48	1.11	0.55	2.24	0.78	0.34	1.77
	Other	0.83	0.51	1.36	2.39	1.22	4.68	1.63	0.76	3.50
**Wealth (ref: Lowest)**										
	Lower	0.79	0.40	1.55	0.38	0.15	0.98	0.87	0.42	1.80
	Middle	1.29	0.60	2.78	0.53	0.20	1.40	1.22	0.63	2.37
	Higher	1.27	0.67	2.40	0.94	0.42	2.12	0.92	0.40	2.10
	Highest	1.35	0.74	2.44	1.03	0.47	2.24	1.27	0.62	2.61
**Source of method (ref: Hospital/Health Center**										
	Health Post/HEW	0.76	0.49	1.17	0.73	0.39	1.37	0.59	0.36	0.98
	Non-public	0.42	0.26	0.69	0.56	0.30	1.02	0.23	0.12	0.44

Adjusted results are shown in Table 4. Results were largely consistent after adjustment. Injectable and other method users were significantly less likely than implant users to be told only about only a bleeding side effect and other method users were significantly less likely to be told about both a bleeding and non-bleeding side effect. Women of higher parity remained significantly more likely to be told about both a bleeding and non-bleeding side effect. Women with secondary education and higher were more likely to receive counseling on bleeding changes only or on both bleeding-and non-bleeding changes.

**Table 4:** Adjusted multinomial logistic regression results identifying the relative risk of receiving counseling on Contraceptive Induced Menstrual Bleeding Changes (CIMBC) only, receiving counseling on non-CIMBC only, or on receiving counseling on both types of side effects, relative to receiving no counseling on side effects. PMA Ethiopia 2019 cross-sectional survey.

		CIMBC only			non-CIMBC only			Both		
		RRR	95% CI		RRR	95% CI		RRR	95% CI	
**Method (ref: implant)**										
	Injectable	0.65	0.46	0.93	0.74	0.42	1.28	0.76	0.50	1.15
	Other	0.31	0.16	0.61	0.81	0.42	1.57	0.43	0.19	0.93
**Parity (ref: 0)**										
	1–2	1.53	0.74	3.17	1.03	0.51	2.09	2.48	1.17	5.29
	3+	1.70	0.79	3.65	1.36	0.64	2.88	2.53	1.15	5.59
**Education (ref: None)**										
	Primary	1.17	0.77	1.78	1.32	0.75	2.31	1.18	0.78	1.78
	Secondary	1.62	1.01	2.62	1.72	0.90	3.32	2.47	1.44	4.21
**Residence (ref: rural)**										
	Urban	0.88	0.54	1.42	0.69	0.37	1.32	1.11	0.61	2.02
**Region (ref: Oromiya)**										
	Tigray	0.40	0.24	0.69	1.00	0.49	2.03	0.87	0.50	1.52
	Amhara	1.03	0.54	1.99	1.44	0.63	3.28	1.34	0.64	2.82
	Addis	0.60	0.32	1.12	0.75	0.34	1.66	1.10	0.57	2.11
	SNNP	0.67	0.33	1.33	1.00	0.49	2.06	0.68	0.29	1.57
	Other	0.86	0.53	1.37	2.19	1.12	4.28	1.78	0.77	4.10
**Source of method (ref: Hospital/Health Center**										
	Health Post/HEW	0.92	0.60	1.40	0.94	0.49	1.81	0.68	0.40	1.13
	Non-public	0.49	0.30	0.82	0.49	0.27	0.90	0.27	0.13	0.55

## Discussion

We find that most contraceptive users in Ethiopia do not receive sufficient counseling, as approximately three in four users reported they were either not counseled on any side effects or received counseling on only one side effect. Women were most likely to receive counseling on contraceptive-induced menstrual bleeding changes, if they received any counseling at all, and few women received counseling on any non-bleeding changes. Our findings highlight the ongoing need for improvements in access to high-quality, comprehensive contraceptive services.

Despite current and recent contraceptive users in Ethiopia experiencing a wide range of side effects [15], fewer than 20% of women reported being counseled on at least two side effects, and the majority of women who received counseling only received counseling on contraceptive-induced menstrual bleeding changes. While concerns about bleeding changes do feature heavily in women’s worries related to contraceptive side effects [14], given the range of potential side effects associated with hormonal contraception and the non-hormonal IUD, this limited counseling does not represent sufficient information exchange between providers and clients. Evidence suggests that women learn about side effects, both clinically validated and not, from a range of sources [31–33], but largely trust providers to deliver scientifically sound and accurate information [9,34]. If women are already aware of and concerned about potential side effects, receiving counseling from providers on the full range of potential side effects is an important opportunity to address concerns and dispel myths. The Federal Ministry of Health of Ethiopia recently introduced a strategy to improve counseling through the use of structured counseling tools in public health facilities, but implementation was at the onset at the time of this publication. Our findings, highlighting where additional counseling may be particularly important, may be useful in informing on-going efforts to refine and implement this tool. Additionally, we found modest differences across regions in counseling patterns. Implementation of a standardized tool across all public health facilities may aid in reducing regional disparities in access to information.

Receipt of more comprehensive counseling was influenced by method choice. On the whole, implant users were the most likely to receive counseling on side effects and pill users were the least likely. While side effect profiles differ by method and thus, counseling should also differ, all hormonal methods have the potential for both bleeding and non-bleeding changes. As the implant requires clinical intervention to stop use, it is potentially positive that implant users received relatively more counseling on side effects, however, all women, regardless of the method they select, should be comprehensively counseled in order to make an informed choice. As with directive counseling based on patient characteristics, differences in counseling by method may be explained by provider concerns about safety or preferences for specific methods [35]. This finding further reinforces the need for research with providers to understand how knowledge of and concerns about side effects influence counseling. Additionally, we found that women who were more educated and those who had a greater number of children were more likely to receive counseling on at least one potential bleeding change and one non-bleeding change related to their contraceptive method. While our study is unable to explain what specific behaviors would influence why nulliparous women would receive less comprehensive counseling, fears related to contraception may be particularly relevant to address among younger and nulliparous women as they tend to have the lowest rates of contraceptive use among women who wish to prevent pregnancy [36,37]. Provider bias towards specific methods based on patient characteristics, such as marital status and parity, may influence counseling, as has been documented elsewhere [9,10,35,38]. Additional research among providers is necessary to understand how user characteristics influence providers’ counseling practices on side effects.

Method-specific differences in counseling experiences may in part be explained by method source. For example, pill users may be more likely to source their method from a range of sources, including pharmacies, and be less likely to rely on public sources. This relationship between method type and counseling remained, however, even after adjusting for method source. Women who received their methods outside of the public health sector had significantly lower relative risk of receiving counseling on any side effects. The majority of women in Ethiopia access their methods through the public health sector [20], but efforts are underway to expand the private sector in Ethiopia through such projects as the Ethiopia Private Health Sector Project [39,40]. Evidence suggests that private facilities are less likely to have trained FP providers or clear guidelines and protocols for FP provision [41]. Thus, expansion of the private sector must be done with quality in mind, ensuring that private providers, particularly drug shop owners and pharmacists, are trained to provide comprehensive counseling. Lessons learned from the implementation of the structured counseling tool, mentioned above, may be particulary useful in designing additional interventions to improve counseling at private facilities.

Our study is not without limitations. Our survey question about contraceptive side effects did not distinguish what information was provided about each side effect or confirm if scientifically accurate information was shared. Such nuance is likely not possible in a population-based survey and requires alternative data collection strategies, such as observation of counseling sessions. Additionally, we collected information retroactively; recall of specific information is likely to fade over time and introduce bias. If so, we may be underestimating the percentage of women who received more comprehensive counseling. However, retrospective recall of contraceptive services is the standard methodology used by PMA, DHS, and MICS. Though there are documented limitations to its use [13,42], it remains a valuable source of information as what women recall is likely to influence their behavior and inform their perceptions of quality [43]. Additionally, given the extremely low percentages of women who report being told about side effects other than contraceptive induced menstrual bleeding changes, such side effects are likely not being adequately addressed during counseling, even if these are underestimates. Similarly, we collect information only among current users of contraception. Women who discontinued their method prior to the survey or who received counseling but chose not to adopt a method may have received different counseling than women who continued, which is not reflected in our findings due to data limitations. Finally, data were collected in Tigray in 2019 prior to the onset of widespread civil conflict and are not able to be generalized to the current situation. Despite these limitations, we believe that the use a high-quality, nationally representative data source that incorporated detailed information on side effect counseling provides clear evidence of gaps in counseling that must be addressed to enhance informed decision-making and improve reproductive health outcomes.

## Conclusions

Women in Ethiopia do not receive sufficient information on side effects during contraceptive counseling, particularly related to side effects outside of menstrual bleeding changes. Private health facilities appear to be of particular concern. Efforts to improve counseling, such as structured counseling tools, should be incorporated into the private sector. Additionally, research with providers is necessary to understand and address biases towards specific methods and counseling messages.

## Supporting information

S1 Checklist STROBE statement—checklist of items that should be included in reports of *cross-sectional studies*. (PDF)
